# The Alteration of Pro-inflammatory Cytokines and Oxidative Stress Markers at Six-Month Post-living Kidney Donation

**DOI:** 10.3389/fmed.2020.00382

**Published:** 2020-07-29

**Authors:** Elodia Nataly Díaz-De la Cruz, José Ignacio Cerrillos-Gutiérrez, Andrés García-Sánchez, Jorge Andrade-Sierra, Ernesto Germán Cardona-Muñoz, Enrique Rojas-Campos, Eduardo González-Espinoza, Alejandra Guillermina Miranda-Díaz

**Affiliations:** ^1^Department of Physiology, Institute of Experimental and Clinical Therapeutics, University Center of Health Sciences, University of Guadalajara, Guadalajara, Mexico; ^2^Department of Nephrology and Transplants, Specialties Hospital, National Occidental Medical Centre, The Mexican Social Security Institute, Guadalajara, Mexico; ^3^Kidney Diseases Medical Research Unit, Specialties Hospital, National Occidental Medical Centre, Mexican Social Security Institute, Guadalajara, Mexico

**Keywords:** kidney living donor, renal transplantation, chronic kidney disease, oxidative stress, oxidative DNA damage

## Abstract

Donors have a higher risk of developing chronic kidney disease than the general population. Some mechanisms mediated by pro-inflammatory cytokines and oxidative stress may be involved as risk factors. The objective of the study was to evaluate the behavior of pro-inflammatory cytokines and oxidative stress markers in living renal donors with a 6-month follow-up. A single prospective cohort was performed in 88 renal donors. At the end of the follow-up, the levels of lipoperoxides, 6.52 ± 1.12 mM, and 8-isoprostanes, 63.75 ± 13.28 pg/mL, were lower than before donation, 10.20 ± 3.95 mM (*p* < 0.001) and 67.54 ± 9.64 pg/mL (*p* = 0.026), respectively. Initial levels of nitric oxide (NO), 356.09 ± 59.38 μM increased at the end of the follow-up, 467.08 ± 38.74 μM (*p* < 0.001). It was observed in the final determination of donors decreased activity of antioxidant enzymes superoxide dismutase (SOD), 0.74 ± 0.57 U/L and glutathione peroxidase (GPx), 556.41 ± 80.37 nmol, in comparison with the levels obtained in the initial determination, 1.05 ± 0.57 U/L (*p* < 0.001) and 827.93 ± 162.78 nmol (*p* < 0.001), respectively. The pro-inflammatory cytokines, Tumor necrosis factor alpha and interleukin-6 showed no differences at 6 months after donation. The enzyme oxoguanine glycosylase (hOGG1) responsible for repairing oxidative damage to DNA, showed a decrease in its concentration at the end of the study in donor men, 0.40 ± 0.21 ng/mL compared to the initial levels, 0.55 ± 0.32 ng/mL (*p* = 0.025). The marker, 8-hydroxy-2-deoxyguanosine (8-OHdG) exhibited an increase in donor men at the final determination 2.28 ± 1.99 ng/mL, compared to the concentration before donation, 1.72 ± 1.96 ng/mL (*p* < 0.001). We found significant changes in the markers of the oxidative state with increased NO and 8-OHdG, as well as a significant decrease in the antioxidant defenses SOD, GPx, and in the DNA repair enzyme in living renal donors after 6 months of follow-up.

## Introduction

Renal transplantation (RT) is the optimal treatment to improve survival and quality of life in patients with end-stage renal disease (ESRD). RT is more profitable and provides a better quality of life and better long-term survival compared to dialysis (1). Renal donors are generally well-selected before donation. Routine clinical parameters (blood pressure, urinary protein, kidney function, mass/body size at the time of donation) have not been strong predictors of long-term risk in donor populations (2). In some countries, living kidney donation is the most important source of organs given the scarcity of kidneys from deceased donors. Increasing confidence, based on satisfactory short and long-term results from living kidney donors, has led to the inclusion of borderline donors (those with older age, hypertensive in control, low-grade proteinuria, and overweight people) (3). Living kidney donor transplantation is an increasingly used treatment for ESRD because it is considered a safe procedure for donors and gives excellent results to transplant recipients. The short and long term safety of donors is paramount to the continued success of living donation (4). However, the donation process is not free of complications; major complications have been reported in 6% and minor in 22% of living donors (5). Even perioperative mortality after nephrectomy in living donors occurs in 3 out of 10,000 donations. In 2014, the possibility of presenting an increased risk of developing ESDR was reported in living renal donors, especially those with factors such as race, age, body mass index (BMI), and decreased glomerular filtration rate (GFR) (6).

There is scarce information related to the behavior and possible role of pro-inflammatory cytokines and oxidative status before and in the long term after renal donation. In 2012 Yap et al. informed an increase in interleukin 6 (IL-6), tumor necrosis factor alpha (TNF-α), and the enzyme aspartate aminotransferase (AST) 24 h after donation *vs*. the results obtained before the kidney donation procedure. Furthermore, the authors report that oxidative stress was higher in living donors before and after surgery than surgeries without nephrectomy (7).

Oxidative stress (OS) is related to the development of complications after organ transplantation by triggering ischemia-reperfusion injury and delayed graft function (8). Additionally, the role of OS in the evolution of ESRD has been reported previously: There is a depletion in endogenous and exogenous antioxidants associated with abnormal protein function and the subsequent ability to trigger damage to deoxyribonucleic acid (DNA) and the possibility to produce irreversible cell damage (9, 10).

It has been reported in the post-surgical period of renal donors increase in lipoperoxidation (LPO) products and a decrease in the activity of antioxidant enzymes, so the presence of oxidative stress (11). The reduction of antioxidant activity in RT may also contribute to oxidative damage to DNA (12). 8-hydroxy-2′-deoxyguanosine (8-OHdG) is a sensitive and specific marker of oxidative damage to DNA with the ability to affect the genome of the individual. The main repair enzyme for oxidative DNA damage is 8-oxoguanine glycosylase (hOGG1), whose preponderant function is produced through mechanisms by base cleavage (13, 14).

The aim of the study was to describe and compare the evolution of pro-inflammatory and oxidative status in living renal donors with a 6-month follow-up.

## Materials and Methods

### Subjects

A prospective cohort study with a 6-month follow-up was performed. Living kidney donors of any gender, >18 years, were included. Donors with a history of consuming over-the-counter or prescription antioxidants 6 months prior to the start of the study were excluded (dietary antioxidants were not evaluated). All donors were from the Department of Nephrology, Transplant Division, at the UMAE Hospital de Especialidades, Centro Médico Nacional de Occidente. Instituto Mexicano del Seguro Social (IMSS) in Guadalajara, Jalisco, Mexico.

### Data Collection

All information was obtained 24 h before the donation date. Clinical information was: gender, age, weight, height, systolic, and diastolic blood pressure, and type of donor (related/unrelated). Biochemical data includes hemoglobin, glucose, urea, creatinine, uric acid, cholesterol, triglycerides, bilirubin, aspartate aminotransferase (AST), alanine aminotransferase (ALT), sodium, potassium, chloride, phosphorus, magnesium, 24 h urine protein and eGFR using CKD-EPI formula. There were two evaluations: baseline and at the end of the follow-up.

### Oxidative Stress Markers

Additionally, two blood samples were taken (5 mL in a dry tube and 5 mL in 0.1% of ethylenediaminetetra-acetic acid) to measure oxidative stress markers and inflammation cytokines. Blood samples were centrifuged to obtained serum and plasma. Samples were stored at −80°C until processing. For the determination of oxidative stress in donors, there were measured the markers of oxidative damage to lipids (lipoperoxides and isoprostanes) and to DNA (8-OHdG and the DNA repair enzyme hOGG1). The oxidizing activity of the endothelium was determined with the measurement of ON. To know the antioxidant capacity of donors, we quantified the activity of the main antioxidant enzymes SOD, GPx, and total antioxidant activity. The overall inflammatory response was measured with the levels of the pro-inflammatory cytokines TNF-alpha and IL-6.

### Products of Lipoperoxidation (LPO)

The colorimetric assay kit no. FR12 for Lipid peroxidation (Oxford Biomedical Research Inc®, Oxford, MI, USA) was performed. Six hundred fifty microlitre of N-methyl-2-phenylindole in acetonitrile and 150 μL of 12 N HCl were added to 200 μL of standards or samples. After vortex and mix, standards, and samples were incubated at 45°C for one h. The clear supernatant was separated from centrifuge samples at 15,000 × g for 10 min. The color was read at 586 nm. Intra assay coefficient of variation (CV) for the duplicate standard was 2.88%.

### 8-Isoprostane (8-IP)

Plasmatic 8-IP was measured by means Abcam following manufacturer s instructions (Abcam ab175819®, Cambridge, United Kingdom). A microplate pre-coated with a capture antibody for 8-IP was used. To each well, 100 μL of standard or sample was added, followed by 100 μL of HRP-conjugate. The plate was incubated two h at room temperature. After washing, 200 μL of TMB substrate was added and incubated for 30 min. The reaction was stopped by adding 2 N sulfuric acid. The plate was read at 45 nm. Intra assay CV for the duplicate standard was 3.77%.

### Nitric Oxide (NO)

Plasma samples were deproteinized by the addition of 6 mg of zinc sulfate to 400 μL of the sample. Then, samples were centrifuged at 10,000 × g for 10 min at 4°C to separate the supernatant. The colorimetric kit NB98, Oxford Biochemical®, Oxford, MI, USA, was used. Ten μL of nitrate reductase, ten μL of 2 mM NADH, and 85 μL of standard or sample were added to each well of a microplate. The reaction was left for 20 min, and 50 μL of two different dyes were added. The plate was shaken for 5 min and read at 540 nm. Intra assay CV for the duplicate standard was 5.63%.

### Antioxidants

#### Superoxide Dismutase (SOD)

SOD activity was measured using method No. 706002 of Cayman Chemical Company®, USA. Serum samples were diluted 1:2 in the buffer sample. Tetrazolium salt was used as a radical's detector. Samples, standards, and radical's detectors were added to a 96 well-microplate. Xanthine oxidase was added, and the plate was incubated 20 min at room temperature. Optical density was read at 440 nm. The duplicate intra-assay standard CV was 2.46%.

#### Glutathione Peroxidase (GPx)

The assay was performed as per the colorimetric kit ab102530 (Abcam®, Cambridge, United Kingdom). Fifty microlitre of plasma samples or standards were added into 96 wells microplate with a mixed solution of NADPH, glutathione reductase, and glutathione. After 15 min of incubation at room temperature, a first lecture was taken at 340 nm, ten microlitre of cumene hydroperoxide was added. A second read was done at 340 nm. The intra-assay for duplicate standard CV was 3.36%.

#### Total Antioxidant Capacity (TAC)

The measure of TAC was performed like the instructions of the kit manufacturer (Total Antioxidant Power Kit, No. TA02.090130, Oxford Biomedical Research®). Serum samples and standards were diluted 1:40, and 200 μL of each was added to a microplate. The plate was read at 450 nm as a reference value. Then 50 μL of the copper solution was added, and the plate was incubated at room temperature for 3 min. The reaction was stopped with 50 μL with a solution containing EDTA. The color development was read at 450 nm. Concentration is expressed as mM Trolox (an analog of vitamin E) equivalents. The intra-assay for duplicate standard CV was 2.13%.

### 8-hydroxy-2′-deoxyguanosine (8-OHdG)

A competitive ELISA kit assay was performed (No. ab201734 Abcam®, Cambridge, United Kingdom). An anti-8-OHdG pre-coated 96-well-plate was used. Fifty μL of serum sample or standard was added to each well with 50 μL of horseradish peroxidase (HRP) conjugated 8-OHdG antibody. The plate was incubated for one h at room temperature. TMB substrate was used to obtain a color signal measured at 450 nm. The intra-assay for duplicate standard CV was 6.04%

### 8-oxoguanine-DNA-N-glycosylase-1 (hOGG-1)

The assay was performed following the ELISA kit hOGG-1 MBS702793 (My Biosource®, San Diego, CA). Briefly, 100 μL of plasma and standards were added to the wells, and the plate was incubated at 37°C for two h. One-hundred microlitre biotinylated antibody was added and incubated at 37°C for one h. Afterward, 100 μL HRP-avidin was added and incubated for one h at 37°C. TMB was used as a substrate. The optical density was read at 450 nm with a correction set at 540 nm. The intra-assay for duplicate standard CV was 10%. For all the optical density measures, the spectrophotometer Synergy^TM^ HT multi-detection microplate reader (Bio-Tek, Winooski, VT, USA) was used.

### TNF-α and IL6

The assay for TN-α and IL-6 in plasma was done using the ELISA kit 900-K25 and 900-K16, respectively (Peprotech®, Rocky Hill, NJ 08553, USA). A 96 well plate was coated for TNF-α or IL-6. Bovine serum albumin 1% in phosphate-buffer was used. The plate was incubated with 100 μL of the sample of standard for two h at room temperature. After washing the plate, 100 μL of capture antibody was added and incubated for two h at room temperature. The plate was washed, and 100 μL of HRP-avidin conjugated was added. ABST solution was used as a substrate. Color development was measured at 405 nm with wavelength correction set at 650 nm. Intra assay coefficient of variation for the duplicate standard was 4.51% for TNF-α and 5.65% for IL-6

### Statistical Analysis

Quantitative data are expressed as mean ± SD or percentages. Qualitative data are reported as frequency and percentages. Kolmogorov-Smirnov Goodness-of-Fit test was used to know the normal distribution of data. The intra-group comparison was conducted with paired Student's-*t* test, or Wilcoxon matched pairs test, *p* ≤ 0.05 was considered statistically significant, with a confidence interval of 95%. SPSS for Windows version 20.0 (IBM SPSS statics Inc., Chicago, IL, USA) was used.

### Ethical Considerations

The research complies with the ethical principles for medical research in human beings stipulated in the Declaration of Helsinki 64th General Assembly, Fortaleza, Brazil, October 2013. In addition to adhering to the standards of Good Clinical Practices. All procedures were performed according to national regulations stipulated in the General Health Legal Guidelines for Health Care Research in Mexico, 2nd Title, in Ethical Aspects for Research in Human Beings, Chapter 1, and Article 17. All patients gave and signed the informed consent form in the presence of signed witnesses. The study was evaluated and approved by the Local Ethics and Research Committee at the *Centro Médico Nacional Del Baj*í*o*, IMSS (R-2018-1001-150).

## Results

Eighty-eight living renal donors were evaluated; the mean age was 35 years old. We found an increase in BMI and a decrease in systolic blood pressure. Serum creatinine concentrations were increased between the initial determination 0.80 ± 0.15 mg/dL and final 1.11 ± 0.23 (*p* = 0.001). The initial uric acid was 278.84 ± 141.19 mg/dL front to 354.57 ± 88.16 mg/dL (*p* = 0.001) in the final determination. Cholesterol also increased, the initial concentration was 132.78 ± 74.76 mg/dL vs. final concentration 179.81 ± 35.55 mg/dL (*p* = 0.001). Triglycerides showed the same behavior, initial 87.73 ± 64.88 mg/dL vs. 145.26 ± 74.92 mg/dL (*p* = 0.001) in the final determination. GFR showed a significant decrease between the result prior to donation of 107.79 ± 17.26 mL/min/1.73m^2^ and the final determination 78.89 ± 19.55 mL/min/1.73m^2^ *(p* < 0.001). Twenty hours urine proteinuria was found to increase significantly at the end of the study with 0.36 ± 0.41 vs. 0.28 ± 0.13 g/24 h at the initial (*p* = 0.013) Table 1.

**Table 1:** Anthropometric and biochemical data of living donors of kidney.

**Age years**	**38.15 ± 10.85**		
**Gender**			
Male (%)	43 (49%)		
Female (%)	45 (51%)		
	**Initial**	**6 months**	***p***
BMI Kg/m^2^	26.29 ± 3.23	26.88 ± 3.97	0.018^*^
Systolic blood pressure mmHg	117.27 ± 9.38	112.87 ± 13.45	0.008^**^
Diastolic blood pressure mmHg	77.71 ± 7.05	77.72 ± 11.70	0.866
Hemoglobin mg/dL	14.75 ± 1.81	14.24 ± 1.79	0.491
Glucose mg/dL	89.70 ± 12.79	91.82 ± 8.39	0.624
Urea mg/dL	30.79 ± 8.57	32.50 ± 7.56	0.141
Creatinine mg/dL	0.80 ± 0.15	1.11 ± 0.23	<0.001^*^
Uric acid μmol/L	278.84 ± 141.19	354.57 ± 88.16	<0.001^**^
Cholesterol mg/dL	132.78 ± 74.76	179.81 ± 35.55	<0.001^*^
Triglycerides mg/dL	87.73 ± 64.88	145.26 ± 74.92	<0.001^**^
Total bilirubin mg/dL	0.71 ± 0.39	0.55 ± 0.28	0.061
AST U/L	24.74 ± 12.31	26.09 ± 11	0.978
ALT U/L	36.76 ± 18.72	26.01 ± 16.71	<0.001^**^
Sodium mmol/L	140.34 ± 14.28	137.50 ± 17.74	0.003^**^
Potassium mmol/L	4.22 ± 0.36	4.39 ± 0.68	<0.001^**^
Chlorine mmol/L	103.73 ± 2.29	105.60 ± 2.28	<0.001^**^
Calcium mg/dL	9.45 ± 0.70	9.42 ± 0.41	0.25
Phosphorus mg/dL	4.06 ± 1.52	3.61 ± 0.57	<0.001^**^
Magnesium mmol/L	0.80 ± 0.09	0.78 ± 0.17	0.985
24 h urine protein g/24-h	0.28 ± 0.13	0.36 ± 0.41	0.013^**^
GFR mL/min/1.73m^2^	107.79 ± 17.26	78.89 ± 19.55	<0.001^*^

*Values are expressed in average ± standard deviation*.

* *Student's t test*.

** *Wilcoxon test*.

*BMI, Body mass index; BP, blood pressure; AST, Aspartate aminotransferase; ALT, Alanine aminotransferase; GFR, Glomerular filtration rate*.

### Oxidative Stress Markers

#### LPO, 8-IP, NO, 8-OHdG, hOGG1

The levels of LPO and 8-IP at 6-months follow-up of organ donation showed a significant decrease *p* < 0.001 and *p* = 0.026, respectively. However, the NO levels between the initial 356.09 ± 59.38 μM and final determination were increased with 467.08 ± 38.74 μM (*p* < 0.001). The 8-OHdG was significantly over-expressed in the final determination 2.28 ± 1.99 ng/mL compared to initial levels 1.72 ± 1.96 ng/mL. Contrary to the DNA repair enzyme level hOGG1 showed significant down-regulated in the final determination with 0.40 ± 0.21 ng/mL compared to initial levels, 0.55 ± 0.32 ng/mL Table 2.

**Table 2:** Oxidative stress markers.

	**Initial**	**6 months**	***p***
**OXIDANTS**			
LPO mM	10.20 ± 3.95	6.52 ± 1.12	<0.001^**^
NO μM	356.09 ± 59.38	467.08 ± 38.74	<0.001^**^
8-IP pg/mL	67.54 ± 9.64	63.75 ± 13.28	0.026^*^
**MARKER OF OXIDATIVE DAMAGE TO DNA**			
8-OHdG ng/mL	1.72 ± 1.96	2.28 ± 1.99	<0.001^**^
hOGG1 ng/mL	0.55 ± 0.32	0.40 ± 0.21	0.025^**^
**ANTIOXIDANTS**			
SOD U/L	1.05 ± 0.57	0.74 ± 0.57	<0.001^**^
GPx nmol	827.93 ± 162.78	556.41 ± 80.37	<0.001^**^
TAC mM	23.49 ± 7.72	25.42 ± 20.69	0.762
**PRO-INFLAMMATORY CYTOKINES**			
IL-6 pg/mL	58.03 ± 16.19	56.34 ± 13.99	0.357
TNF- α pg/mL	162.94 ± 50.77	173.29 ± 130.17	0.513

*Values are expressed in average ± standard deviation*.

* *Student t test*.

** *Wilcoxon test*.

*LPO, lipoperoxides; NO, nitric oxide; 8-IP, 8-Isoprostane; 8-OHdG, 8-hydroxy-2′-deoxyguanosine; hOGG1, oxoguanine glucosidase; SOD, Superoxide dismutase; GPx, glutathione peroxidase; TAC, total antioxidant capacity; IL-6, interleukin 6; TNF-α, tumor necrosis factor alpha*.

### Antioxidants

The antioxidant activity of SOD, 0.74 ± 0.57 U/L and GPx 556.41 ± 80.37 nmol decreased significantly in the final determination (*p* < 0.001) compared to the initial determination 1.05 ± 0.57 U/L for SOD and 827.93 ± 162.78 nmol for GPx. No changes were found in the levels of total antioxidant capacity Table 2.

### Pro-inflammatory Cytokines

Pro-inflammatory cytokines showed similar results between initial IL-6 determination, 58.03 ± 16.19 pg/mL, and final 56.34 ± 13.99 pg/mL (*p* = 0.357). TNF-α results were also similar between initial and final levels 162.94 ± 50.77 pg/mL vs. 173.29 ± 130.17 pg/mL (*p* = 0.513) Table 2.

Results were separated by gender of living kidney donors to determine data behavior. BMI increased at 6 months follow-up with 26.95 ± 4.03 kg/m^2^ (*p* = 0.012) and cholesterol concentration at the end of follow-up in women at 184.27 ± 35.08 mg/dL (*p* < 0.001) were found. Creatinine, uric acid, and triglycerides were found to be significantly increased in both genders (*p* < 0.001). GFR decreased significantly in both genders, predominantly in men (*p* < 0.001) at 72.79 ± 16.41 mL/min/1.73m^2^ and in women at 82.89 ± 20.62 mL/min/1.73m^2^ (*p* < 0.001) Table 3.

**Table 3:** Data according to the gender of renal donors.

	**Male**			**Female**		
	**Initial**	**6 months**	***p***	**Initial**	**6 months**	***p***
Weight kg	77.92 ± 11.26	77.50 ± 13.12	0.902	67.14 ± 8.37	70.92 ± 12.37	0.005^**^
BMI Kg/m^2^	26.27 ± 3.33	26.80 ± 3.96	0.418	26.30 ± 9.96	26.96 ± 4.03	0.012^*^
PA systolic mmHg	120.58 ± 9.11	116.06 ± 13.44	0.058	114.11 ± 8.61	110.10 ± 12.99	0.07
PA diastolic mmHg	80.00 ± 6.1	79.38 ± 10.28	0.604	75.53 ± 7.27	75.05 ± 12.61	0.467
Hemoglobin mg/dL	15.73 ± 1.58	15.86 ± 1.05	0.358	13.83 ± 1.51	13.14 ± 1.28	0.015^**^
Glucose mg/dL	89.45 ± 15.22	91.64 ± 8.42	0.373	89.93 ± 10.18	91.94 ± 8.50	0.843
Urea mg/dL	32.16 ± 6.78	33.96 ± 6.38	0.401	29.31 ± 9.92	31.54 ± 8.20	0.222
Creatinine mg/dL	0.88 ± 0.13	1.30 ± 0.20	<0.001^*^	0.72 ± 0.12	0.99 ± 0.15	<0.001^*^
Uric acid μmol/L	305.75 ± 172.07	406.57 ± 94.65	<0.001^**^	253.73 ± 100.18	319.35 ± 63.77	<0.001^**^
Cholesterol mg/dL	131.80 ± 71.45	173.13 ± 36.1	0.145	133.67 ± 78.81	184.27 ± 35.08	<0.001^**^
Triglycerides mg/dL	89.25±+62.98	160.49 ± 75.77	0.022^**^	86.36 ± 67.25	135.10 ± 73.86	<0.001^**^
Total bilirubin mg/dL	0.86 ± 0.44	0.59 ± 0.20	0.083	0.59 ± 0.28	0.52 ± 0.32	0.301
AST U/L	28 ± 14.81	30.89 ± 13.98	0.304	21.78 ± 8.63	23.09 ± 7.43	0.819
ALT U/L	42.52 ± 21.80	35.47 ± 19.84	0.007^**^	31.38 ± 13.44	20.09 ± 11.15	<0.001^**^
Sodium mmol/L	139.19 ± 20.20	139.62 ± 6.04	0.026^**^	141.44 ± 3.32	136.09 ± 22.35	0.06
Potassium mmol/L	4.28 ± 0.38	4.29 ± 10.03	0.053	4.16 ± 0.32	4.46 ± 0.30	<0.001^**^
Chlorine mmol/L	103.23 ± 1.94	104.71 ± 2.63	0.030^**^	104.20 ± 2.51	106.18 ± 1.84	0.003^**^
Calcium mmol/L	9.47 ± 0.87	9.46 ± 0.36	0.202	9.45 ± 0.48	9.39 ± 0.44	0.81
Phosphorus mmol/L	4.30 ± 0.57	3.56 ± 0.32	<0.001^**^	3.82 ± 2.04	3.66 ± 0.70	<0.05^**^
Magnesium mmol/L	0.80 ± 0.09	0.79 ± 00.19	0.89	0.79 ± 0.08	0.78 ± 0.16	0.927
24 h urine protein g/24-h	0.19 ± 0.14	0.43 ± 0.58	0.07	0.22 ± 0.13	0.30 ± 0.19	0.09
GFR mL/min/1.73m^2^	110.71 ± 17.66	72.79 ± 16.41	<0.001^*^	105.00 ± 16.58	82.89 ± 20.62	<0.001^*^

*Initial data and 6-month follow-up of kidney donation according to gender. Values are expressed on average ± standard deviation*.

* *Student's t test*.

** *Wilcoxon test*.

*BMI, Body mass index; BP, Blood pressure; AST, Aspartate aminotransferase; ALT, Alanine aminotransferase transaminase; GFR, Glomerular filtration rate*.

### Oxidants and Antioxidants 6 Months Follow-Up

The LPO and 8-IP levels in both genders were significantly decreased the latter predominantly in women. NO levels were found to be significantly increased in both genders. In relation to antioxidants, the catalytic activity of the SOD enzyme and GPx were significantly decreased in both genders Table 4.

**Table 4:** Oxidative stress marker according to gender.

	**Male**			**Female**		
	**Initial**	**6 months**	***p***	**Initial**	**6 months**	***p***
**OXIDANTS**						
LPO mM	10.16 ± 3.56	6.58 ± 1.27	<0.001^**^	10.23 ± 4.29	6.46 ± 0.99	<0.001^**^
NO μM	368.83 ± 52.44	474.50 ± 31.07	<0.001^**^	344.67 ± 63.48	460.41 ± 43.85	<0.001^**^
8-IP pg/mL	64.97 ± 9.75	64.04 ± 12.07	0.727	69.65 ± 9.24	63.49 ± 14.60	0.011^*^
**MARKER OF OXIDATIVE DAMAGE TO DNA**						
8-OHdG ng/mL	1.29(0.15–1.82)	1.53(1.28–1.83)	<0.003^**^	1.38(0.20–1.95)	1.41(1.25–1.65)	0.12
hOGG1 ng/mL	0.55 ± 0.35	0.35 ± 0.12	0.035^**^	0.53 ± 0.30	0.44 ± 0.26	0.29
**ANTIOXIDANTS**						
SOD U/L	0.95 ± 0.43	0.69 ± 0.56	0.002^**^	1.14 ± 0.66	0.78 ± 0.57	<0.001^**^
GPx nmol	860.89 ± 161.30	548.54 ± 90.01	<0.001^*^	799.91 ± 170.71	562.90 ± 71.70	<0.001^**^
TAC nM	24.39 ± 7.83	29.30 ± 21.81	0.292	22.70 ± 7.63	22.05 ± 19.30	0.63
**PRO-INFLAMMATORY CYTOKINES**						
IL-6 pg/mL	58.28 ± 10.81	54.42 ± 12	0.096	57.81 ± 17.89	58.01 ± 15.48	0.85
TNF-α pg/mL	161.29 ± 38.41	154.20 ± 34.08	0.445	164.412 ± 60	190.01 ± 174.71	0.39

*Initial and follow-up results of the components of oxidative stress in kidney donors according to gender*.

*Values are expressed in average ± standard deviation and ranges*.

* *Student's t test*.

** *Wilcoxon test*.

*LPO, Lipoperoxides; NO, Nitric oxide; 8-IP, 8-Isoprostane; 8-OHdG, 8-hydroxy-2′-deoxyguanosine; hOGG1, Oxoguanine glycosidase; SOD, Superoxide dismutase; GPx, Glutathione peroxidase; TAC, Total antioxidant capacity; IL-6, Interleukin 6; TNF-α, Tumor necrosis factor alpha*.

### Oxidative Damage to DNA and Pro-inflammatory Cytokines at 6 Months Follow-Up

The levels of the 8-OHdG were found to be significantly over-expressed in men. In an inverse relationship, the DNA repair enzyme showed significant down-regulation in men. Pro-inflammatory cytokines, IL-6, and TNF-α, showed a similar level in both genders between the result prior to kidney donation and at 6-months follow-up Table 4.

## Discussion

Oxidative stress is characterized by the imbalance between the production of oxidants and the consumption of antioxidant defenses present in the body (15). Reactive oxygen species (ROS) are continuously generated during normal processes of cellular metabolism. ROS production is possible by external sources such as ultraviolet radiation, smoke, ozone, chemotherapeutic agents, toxins, etc. (16). Under physiological conditions, the crucial and delicate balance of ROS is maintained by endogenous antioxidants by eliminating free radicals (17). In the present study, we investigated the levels of various oxidants, antioxidants, and markers of oxidative damage to DNA before the procedure (24 h) of surgical donation and at 6 months of follow-up. We found a significant increase in NO levels at the end of the follow-up, which could suggest that the living renal donors were in nitrosative stress. Although NO performs beneficial functions as a messenger and in defense of the host, excessive production of NO has a cytotoxic capacity as a result of the reaction of NO with ROS and reactive nitrogen species (RNS) that leads to the formation of peroxynitrite anions, nitration of tyrosine proteins, and hydroxyl radical production (18, 19). Peroxynitrite formation shows participation in renal ischemia-reperfusion injury (20), diabetic glomerular lesions (21), and acute kidney injury for sepsis (22). The increase of nitration of tyrosine proteins is found in arteries from chronic kidney disease patients, especially in arteries with media calcification (23).

LPOs result from ROS reactions; malondialdehyde is a final compound of the LPO process. Its determination is useful as an indicator of oxidative stress in patients with CKD, where its elimination slows down (24, 25). In the present study, the final determination of LPO in renal donors was found in significantly decreased levels compared to the obtained before donation. 8-IP are products from peroxidation of unsaturated fatty acids like arachidonic acid. 8-IP are potent renal vasoconstrictors, too (26). The oxidative consequences of 8-IPs in endothelial function, vascular tone, and ischemia/reperfusion injury are through the thromboxane A_2_ prostanoid receptor signaling (27). Experimental studies in rats show a selective effect of 8-IP in decreasing renal blood flow and GFR with minimal changes in systemic blood pressure (28). In the present study, we found significantly decreased levels of 8-IP between initial and final determination in living renal donors.

SOD activity emerges as the main endogenous antioxidant enzyme with the ability to neutralize ROS (29). SOD is responsible for the elimination of the superoxide radical anion (${\text{O}}_{2}^{-}$) to avoid altering the endothelial function of the vasculature (29). In this study, the activity of the SOD enzyme in renal donors was reduced at 6 months follow-up.

GPx has the ability to eliminate hydrogen peroxide (H_2_O_2_) formed by the presence of transition metals. H_2_O_2_ reduces the hydroxyl radical (^.^OH) by the metals-catalyzed Haber-Weiss reaction; the ·OH radical is extremely reactive (30). Our findings show a significant decrease in the activity of the GPx enzyme at 6 months follow-up of renal donation. These findings could be related to those mentioned above by Ceballos-Picot et al. who report that the progressive decrease in GPx plasma activity results from the gradual damage of the active part of the nephrons responsible for the biosynthesis of the enzyme (31). We can hypothesize that the significant decrease in the activity of the SOD and GPx enzymes could be due to a compensatory effect due to the significant increase in NO and favor the possibility of nitrosative stress in living renal donors.

8-OHdG is a sensitive marker to free radical damage caused by cellular respiration, cell damage, and exposure to endogenous oxidants. In oxidative damage to DNA, there is the oxidation of bases, hydroxylation of guanine, and breakage of DNA chains manifested by the presence of genetic clasts. Therefore, the finding of over-expression of the 8-OHdG in kidney donor men in the final determination is of paramount importance (32).

In a recent paper, Karahan et al. report that kidney donors exhibit more oxidative DNA damage and less antioxidant activity. The authors found a positive correlation between the serum creatinine pre-donation results with the ratio of the marker 8-OHdG/dG, in addition to a negative correlation with the antioxidant activity of paraoxonase (33). The over-expression of 8-OHdG could be relevant because it has been observed that elevated levels of oxidative stress produce crucial mutations with a propensity for mutagenesis (34). Matsumoto et al. reported in renal recipients before reperfusion and during the post-surgical period that 8-OHdG concentrations showed no association between serum levels and graft functioning (35). The malignancy after RT remains inconsistent. It is suggested that the safety of kidney donation should be carried out by long-term follow-up. In a recent study, Wang et al. reported that RT is associated with an increased risk of cancer during long-term follow-up (36). Mortality data from the United States and other countries have identified cancer as the leading cause of death among living renal donors. Recent mortality records compiled by the Organ Transplantation and Acquisition Network (OPTN) found that cancer is the most common cause of death within 7 years of kidney donation, accounting for 10.3% of overall deaths and 23.8% of deaths with a reported cause (37). The follow-up of the donors included in our study was very short (6 months). We found no malignancy in the donors at the end of the follow-up. However, a 13-year follow-up study was recently reported by Shih-Yi Lin et al. that the general health *status* of living r donors was not inferior to that of non-kidney donors. The results in relation to the risk of metabolic syndrome, ESRD, stroke, acute myocardial infarction, and cancer were similar for both groups. However, living renal donors tended to have a higher incidence rate of acute kidney failure than non-donors (38). Regarding the significant increase in the 8-OHdG in living renal donors of males included in the present study, we did not find any information in the available literature on the prevalence in males. It will be necessary to carry out studies with a larger number of donors with long-term follow-up.

The evaluation of DNA repair mechanisms is becoming increasingly important. It is possible to repair oxidative damage to DNA by specific exonucleases for the 8-OHdG marker (14). In the present study, concordance in the significant decrease in the activity of the enzyme hOGG1 was found primarily relevant in the male gender at 6 months follow-up of renal donation. The decrease in DNA repair enzyme could be due to the compensatory consumption of the significant increase in the 8-OHdG or due to the down-regulation of the enzyme.

Obtaining the sample between female and male donors was similar; for that reason, we considered assessing whether the male or female gender showed any impact. Clinical evidence report that women have higher OS than men (39). However, the impact of gender on OS varies according to the measured markers and the pathophysiological conditions (40). Estrogens are attributed to antioxidant properties to decrease 8-IP levels in premenopausal women compared to healthy men (41). Our study supports the hypothesis that women have a better ability to counteract 8-IP production than men. Cakatay et al. evaluated in a murine model the impact of gender on the levels of several biomarkers of oxidative stress, and they discovered that men obtained higher levels of 8-OHdG than women (42). In another study published in 2002, the authors compared the levels of 8-OHdG between males and females classified as clinically healthy. The authors found that males had elevated levels of certain oxidized bases compared to females (13). In our study, we found that male donors showed an increase in the concentration of 8-OHdG, 6 months after donation. This finding might suggest that the male gender may be more likely to develop ESRD (33, 43). In 2016, lifetime ESRD risk was reported to be up to 50% higher in men compared to women in all racial/ethnic groups. These findings suggest that women may have a slower decline in kidney function compared to men or that men are more likely to die earlier when progressing to ESRD (44).

Inflammatory mediators could measure the response to intraoperative stress; the most sensitive marker that represents surgical stress is IL-6 (45). The levels of pro-inflammatory cytokines we found were similar between the initial determination and the determination at the end of the follow-up. In the present study, pro-inflammatory cytokine levels were performed 24 h before the donation procedure and at 6 months follow-up, so we did not expect changes in the levels of IL-6 and TNF-α. Experimental data suggest that increased OS can lead to proteinuria (46). IL-6 is positively related to the development of albuminuria proteinuria and proteinuria in patients with hematopoietic cell transplant (47). Also, a study shows that TNF-α is an independent predictor of urine protein excretion in diabetic patients (48). The comprehensive information, including inflammation monitoring and oxidative stress markers for the long-term health of living renal donors, is woefully limited.

The quantification of the GFR <60 mL/min/1.73m^2^ has important implications for the definition of CKD in renal donors over time (49). In our study, we found a significant decrease in GFR at 6 months of follow-up, which suggests that renal donors are approximately in stage 2 of CKD (50). Although it has been previously reported that living kidney donors experience an early reduction in GFR of 25–40% after nephrectomy, this decrease could improve in the long term (51). The fundamental principle is close to medical surveillance and the commitment of the donor and his family to maintain a healthy lifestyle, at 6 months of follow-up. Also, we found a significant increase in creatinine, uric acid, cholesterol, triglycerides, and proteinuria. In 2006, it was reported that renal donation produces small increases in proteinuria in 24 h urine and that the initial decrease in GFR does not accelerate in the next 15 years (52). Therefore, the significant increase in serum creatinine and proteinuria in 24-h urine could be explained by the nephrectomy and the short follow-up time of the present study, which could improve with long-term follow-up as reported by Garg AX, Kasiske BL and Gourishankar et al. (52–54).

In conclusion, we found OS imbalance characterized by a significant increase in NO that could suggest long term molecular and genetic changes with unclear implications in living renal donors at a 6-month follow-up. The significant increase in the 8-OHdG marker (predominantly in men) and a significant decrease in the antioxidant enzymes SOD, GPx, and the DNA repair enzyme (the latter predominantly in men), which could suggest long-term molecular and genetic complications term. According to the available literature, we cannot explain the reason why the marker 8-OHdG and the repair enzyme to DNA were found to be predominant in male living renal donors at the end of follow-up.

Among the findings, we highlight the significant decrease in GFR and the increase in creatinine and proteinuria in 24-h urine at the end of follow-up of living renal donors. These changes could be implicitly due to donation nephrectomy. Perhaps these alterations could be recovered over time. Because in the present study, no measurements were made at other times (1, 3 months) of follow-up, we cannot hypothesize the behavior of GFR, serum creatinine, and 24 h proteinuria over time. Other studies with short, medium, and long-term follow-up are required to determine the adaptation period of patients to a single kidney.

We believe that the policies that regulate donor follow-up need to provide a close balanced assessment of living renal donors and ensure the improvement of the kidney function. Recommended care for kidney donors should include the annual measurement of serum creatinine and blood pressure at least once a year, the estimation of GFR, and the periodic measurement of albuminuria throughout the life of the donor.

The limitations of the study are due to the small number of living renal donors included and the short follow-up time. The results reflected in the study are not intended to assume that kidney donation is a causal factor in the oxidative state found. Studies involving more donors with long-term follow-up are required.

Strengths. The present study provides original knowledge on the notable imbalance of the oxidative state detected at only 6 months of follow-up after renal donation.

## Data Availability Statement

Most clinical data is restricted to protect information against misuse. The data may be shared by the Mexican Social Security Institute (IMSS) through the approval of the Ethics Committee of the Institution. The data used to support the findings of this study will then be available through the corresponding author at the request of researchers who meet the criteria for access to confidential data.

## Ethics Statement

The studies involving human participants were reviewed and approved by Local Ethics and Research Committee at the Centro Médico Nacional Del Bajío, IMSS (R-2018-1001-150). The patients/participants provided their written informed consent to participate in this study.

## Author Contributions

ED-D, JC-G, and AG-S: conception and design. JA-S, EG-E, EC-M, and AG-S: analysis and interpretation of data. ER-C, EG-E, and AM-D: drafting the article and revising it critically for important intellectual content. All authors contributed to the article and approved the submitted version.

## Conflict of Interest

The authors declare that the research was conducted in the absence of any commercial or financial relationships that could be construed as a potential conflict of interest.
